# P-1009. What Can We Learn from Bezlotoxumab: A Retrospective Evaluation of Practice Patterns

**DOI:** 10.1093/ofid/ofaf695.1206

**Published:** 2026-01-11

**Authors:** Jacob Denkins, HaYoung Ryu, Amber C Streifel, Jim Lewis

**Affiliations:** OHSU, Portland, Oregon; Oregon Health & Science University Hospital and Clinics, Portland, Oregon; Oregon Health and Science University, Portland, Oregon; OHSU, Portland, Oregon

## Abstract

**Background:**

BEZ is a monoclonal antibody recommended as adjunctive therapy for prevention of recurrent *Clostridioides difficile* infection (rCDI). The greatest reduction in recurrence and 30-day hospitalization is observed in patients with rCDI and ≥ 2 risk factors (age ≥65, immunosuppression, severe CDI). Although BEZ was withdrawn from the market, lessons learned from it may inform future tools to guide the use of microbiota-based therapies in preventing rCDI.

**Figure:**
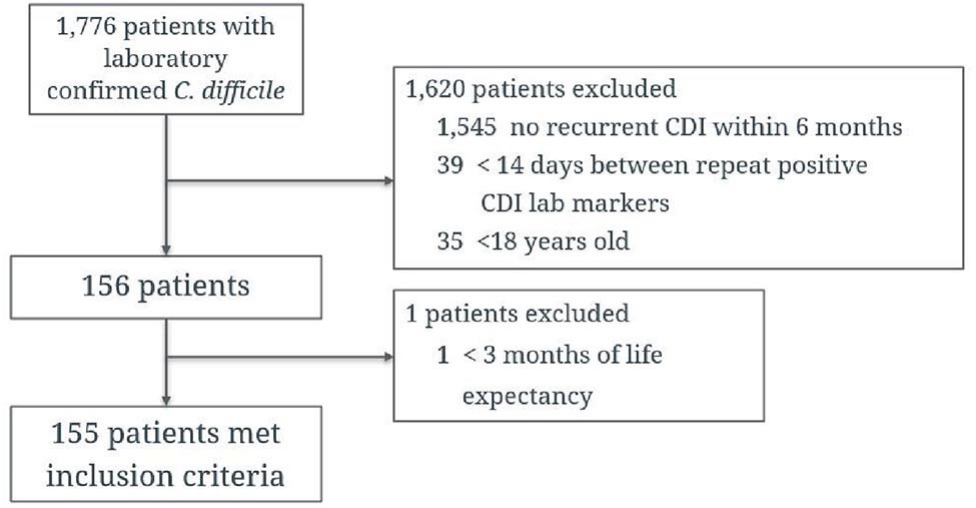
Patient Inclusion Flow Diagram Patient screening and inclusion flow diagram. Of 1,776 patients with laboratory-confirmed C. difficile, 155 met criteria for BEZ eligibility after exclusions.

**Figure:**
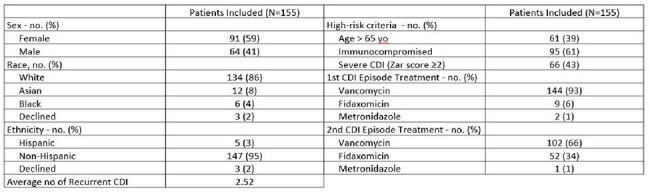
Baseline Characteristics Baseline characteristics of 155 patients eligible for bezlotoxumab (BEZ). Includes demographics, high-risk criteria for recurrent CDI, and treatment regimens for first and second CDI episodes.

**Methods:**

This retrospective cohort study evaluated adult inpatients with laboratory-confirmed rCDI eligible for BEZ at a tertiary academic medical center between March 2018 and July 2024. Patients were included if they were ≥ 18 years of age, had a positive PCR and toxin testing (or indeterminate toxin with clinical symptoms), and had prior CDI within 6 months. The primary objective was to determine the proportion of patients at highest risk for rCDI (≥ 2 risk factors) that did not receive BEZ. Secondary objectives were to compare recurrence rates between patients who did and did not receive BEZ and estimate potential cost avoidance had all highest risk patients received BEZ. Potential cost avoidance was calculated by multiplying the number of highest risk patients by the average number of recurrences and healthcare cost per recurrence, then applying the expected reduction in recurrence with BEZ and subtracting the total drug cost.

**Figure:**
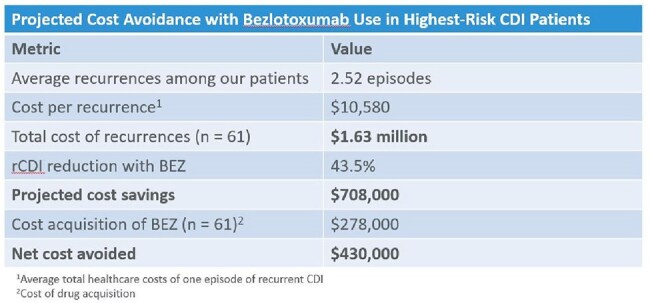

Estimated cost avoidance associated with bezlotoxumab use in 61 highest-risk patients eligible for treatment. Average recurrence burden and recurrence reduction with BEZ were derived from our cohort; cost per recurrence was based on published healthcare cost estimates.

**Results:**

Of 1,776 patients screened, 155 met inclusion criteria for BEZ, including 75 highest risk. Among the highest risk group, 81.3% (61/75) did not receive BEZ. Across all 155 patients eligible for BEZ, rCDI occurred in 46.2% (61/132) of patients not given BEZ compared to 26.1% (6/23) of BEZ recipients. Based on an average total healthcare cost of $10,580 per rCDI episode, treating the remaining 61 highest risk patients could have resulted in $430,000 in net cost avoidance.

**Conclusion:**

Most patients with highest risk of rCDI did not receive BEZ, highlighting a significant missed opportunity to reduce recurrence and associated healthcare costs. This underscores the importance of utilizing available therapies with proven efficacy in preventing rCDI and supports the development of future decision tools to optimize the timely use of microbiota-based interventions.

**Disclosures:**

Jim Lewis, PharmD, FIDSA, Merck: Advisor/Consultant

